# Reissner fibre-induced urotensin signalling from cerebrospinal fluid-contacting neurons prevents scoliosis of the vertebrate spine

**DOI:** 10.1242/bio.052027

**Published:** 2020-05-14

**Authors:** Hao Lu, Aidana Shagirova, Julian L. Goggi, Hui Li Yeo, Sudipto Roy

**Affiliations:** 1 Institute of Molecular and Cell Biology, Proteos, 61 Biopolis Drive, Singapore 138673; 2 Department of Biological Sciences, National University of Singapore, 14 Science Drive 4, Singapore 117543; 3 Singapore Bioimaging Consortium, Helios, 11 Biopolis Way, Singapore 138667; 4 Department of Pediatrics, Yong Loo Lin School of Medicine, National University of Singapore, 1E Kent Ridge Road, Singapore 119288

**Keywords:** Cilia, Cerebrospinal fluid, Reissner fibre, Cerebrospinal fluid-contacting neurons, Urotensin-related peptide, Slow-twitch muscle

## Abstract

Reissner fibre (RF), discovered by the 19^th^-century German anatomist Ernst Reissner, is a filamentous structure present in cerebrospinal fluid (CSF). RF forms by aggregation of a glycoprotein called SCO-spondin (Sspo), but its function has remained enigmatic. Recent studies have shown that zebrafish *sspo* mutants develop a curved embryonic body axis. Zebrafish embryos with impaired cilia motility also develop curved bodies, which arises from failure of expression of *urotensin related peptide* (*urp*) genes in CSF-contacting neurons (CSF-cNs), impairing downstream signalling in trunk muscles. Here, we show that *sspo* mutants can survive into adulthood, but display severe curvatures of the vertebral column, resembling the common human spine disorder idiopathic scoliosis (IS). *sspo* mutants also exhibit significant reduction of *urp* gene expression from CSF-cNs. Consistent with epinephrine in CSF being bound by RF and required for *urp* expression, treating *sspo* mutants with this catecholamine rescued expression of the *urp* genes and axial defects. More strikingly, providing Urp2, specifically in the CSF-cNs, rescued body curvature of *sspo* homozygotes during larval stages as well as in the adult. These findings bridge existing gaps in our knowledge between cilia motility, RF, Urp signalling and spine deformities, and suggest that targeting the Urotensin pathway could provide novel therapeutic avenues for IS.

## Abstract (precis)

**Summary:** Reissner fibre (RF) is a glycoprotein filament suspended in cerebrospinal fluid (CSF). We show that RF regulates zebrafish spine morphogenesis by regulating Urotensin signalling from CSF-contacting neurons of the spinal cord.

## INTRODUCTION

Form and function of the vertebrate body is intimately dependent on proper morphogenesis of the spine. Detrimental effects of spinal abnormalities is best exemplified by the human disorder idiopathic scoliosis (IS), a debilitating disease that manifests in three-dimensional curvatures of the vertebral column, causing disfigurement of the torso, chronic back pain, postural and gait problems as well as breathing difficulties, and affects up to 3% of children and adolescents worldwide (Cheng et al., 2015). The defining feature of IS, lateral curvatures of the spine without obvious malformations of the vertebrae themselves (hence idiopathic), has confounded the discovery of the etiological basis of the disease. Consequently, treatment options for IS are largely limited to wearing corrective braces or invasive surgery, particularly in cases with acute deformations. Using genetic analysis in the zebrafish, we and others have implicated impairment in cilia-driven cerebrospinal fluid (CSF) flow within the brain and spinal canal in the development of spine curvature in the embryo and adult (Grimes et al., 2016; Zhang et al., 2018). In line with this, mutations in *POC5*, encoding a centrosome and ciliary basal body protein, have been associated with IS (Patten et al., 2015; Hassan et al., 2019), suggesting that abnormalities in cilia could also extend to and possibly underlie the pathobiology of the human disease. Downstream of CSF flow, we have shown that epinephrine, transported by CSF, induces the expression of the Urp family of cyclic neuropeptides in CSF-cNs of the zebrafish spinal cord (Zhang et al., 2018). CSF-cNs are postulated to be secretory chemo- and mechanosensory neurons that develop along the spinal canal, with the apical surface of their cell bodies in direct contact with circulating CSF (Orts-Del'Immagine and Wyart, 2017). Urp proteins, secreted from CSF-cNs, likely function via their receptor, Uts2r3, on slow-twitch muscle fibres of the dorsal somites, and the current hypothesis posits that contractile activity of these muscle fibres brings about proper axial morphogenesis (Zhang et al., 2018).

RF is an extracellular thread-like structure that floats in CSF of the brain and spinal canal (Rodríguez et al., 1998). RF has been described from many vertebrate species, including human embryos and an adolescent, although its existence in the adult remains controversial (Olry and Haines, 2003). RF has been variously associated with a wide diversity of developmental and physiological functions of the nervous system, including spiritual consciousness (Grondona et al., 2012; Rodríguez et al., 1998; Wile, 2012); however, definitive evidence for any of these biological roles is lacking. Recently, mutations in Sspo, a very large glycoprotein (>5000 amino acids) secreted from the subcommissural organ (SCO) and floor plate that aggregates to form RF, have been shown to abolish RF biogenesis and also cause ventral body curvature in embryonic zebrafish (Cantaut-Belarif et al., 2018), reminiscent of cilia mutants described previously (Grimes et al., 2016; Zhang et al., 2018). This work also demonstrated that ciliary motility is required to build RF: in cilia mutants, Sspo aggregation fails and RF assembly is impaired (Cantaut-Belarif et al., 2018). Given these considerations, we set out to investigate whether RF influences proper axial development by regulating Urp signalling in CSF-cNs of the spinal cord, downstream of ciliary motility.

## RESULTS

### Zebrafish *sspo* mutants can survive into adulthood and display severely scoliotic spines

**Fig. 1.**
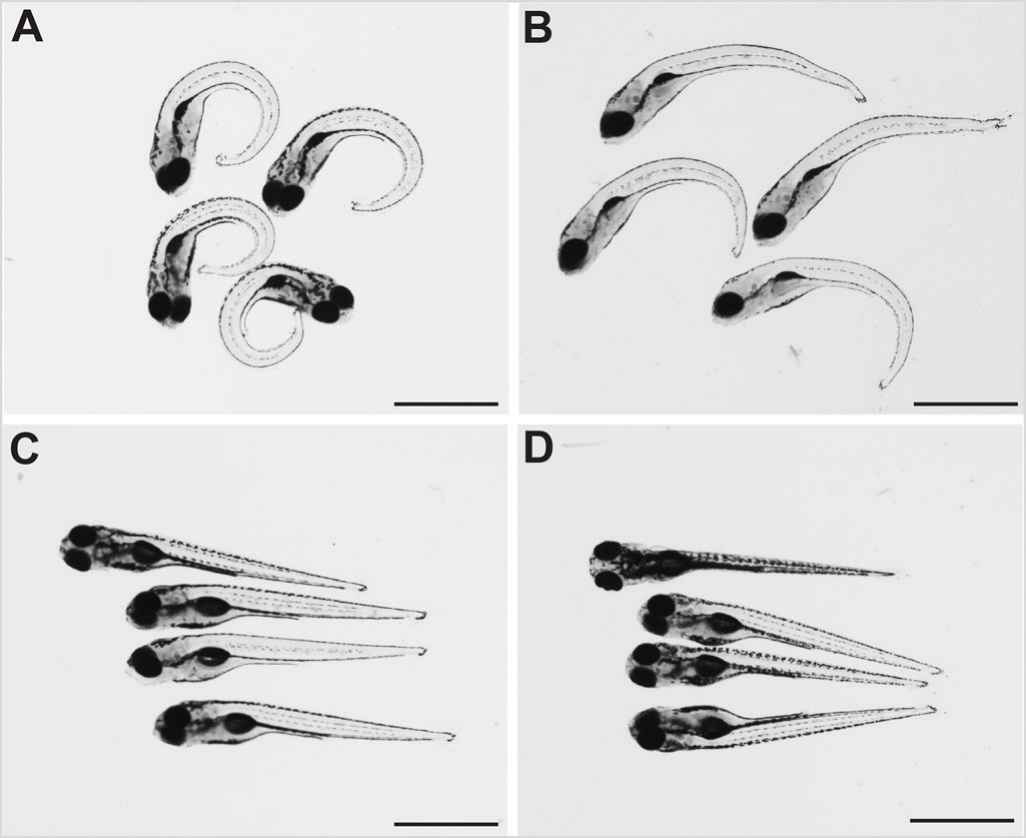
**Dechorionation partly ameliorates the strong ventral curvature of the axis of *sspo* mutants.** (A) *sspo*-mutant larvae that naturally hatched out of the chorion. (B) *sspo*-mutant larvae that were dechorionated at 24 hpf. (C) Wild-type sibling larvae that naturally hatched out of their chorion. (D) Wild-type sibling larvae that were dechorionated at 24 hpf. All larvae shown were imaged at 5 days post-fertilization (dpf) and are representative of 12 embryos analysed for each category. Scale bars: 1 mm.

**Fig. 2.**
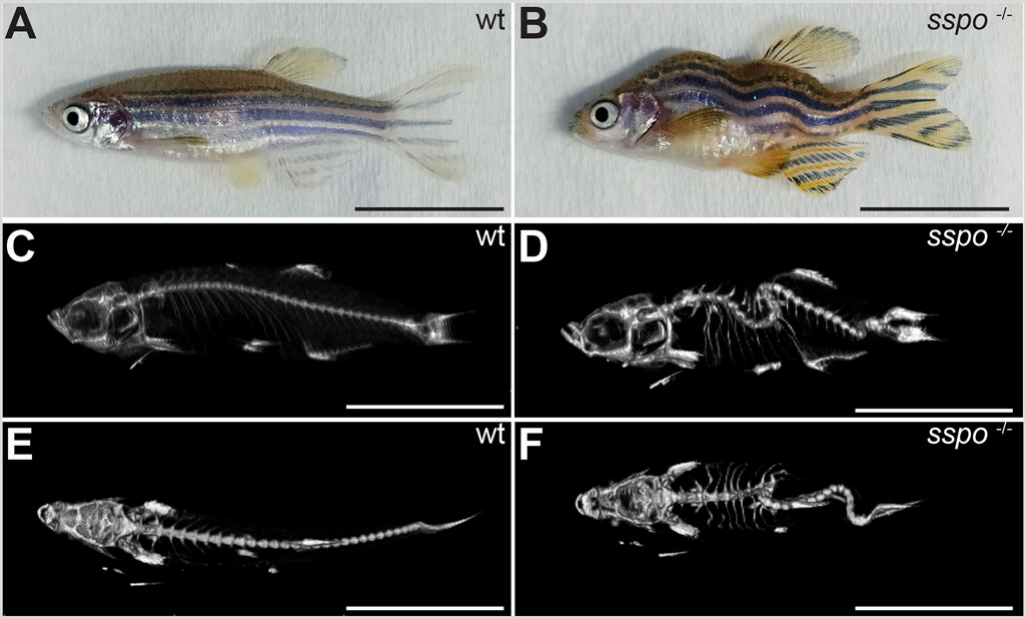
***sspo* mutants develop into adults with scoliotic spines.** (A) A wild-type adult zebrafish. (B) An *sspo* mutant. Note the curved malformations of the trunk and tail. (C) MicroCT scan image of a wild-type zebrafish (lateral view). (D) MicroCT scan image of an *sspo*-mutant zebrafish (lateral view). Note the dorso-ventral curvatures of the spine. (E) MicroCT scan image of the wild-type zebrafish (dorsal view). (F) MicroCT scan image of the *sspo*-mutant zebrafish (dorsal view). Note the lateral curvatures of the spine. All fish were 3 months of age. Two fish were analysed for each genotype. Scale bars: 1 cm.

Zebrafish embryos homozygous for a null allele of *sspo* have been described to be embryonic lethal, possibly due to severe curvature of the axis that precludes proper swimming movements of the mutant larvae (Cantaut-Belarif et al., 2018). We reasoned that the severity of ventral axial curvature could be ameliorated to some degree by releasing the mutant embryos from the confines of the spherical chorion (which induces lateral curvature of the growing axis, and thus could be exacerbating the axial curvature of *sspo* mutants) and this could allow them to develop into adults. Indeed, removal of the chorion at 24 h post-fertilisation (hpf), at the time of onset of ventral curvature of the trunk and tail, produced embryos with a less severely curved axis to varying degrees (Fig. 1A–D), and many (*n*=8, ∼30%) of these precociously dechorionated embryos developed into swimming larvae that subsequently matured into adults. However, the adult fish exhibited strong curvatures of the spine, highly reminiscent of what we and others have documented before for mutations in the Urp receptor, Uts2r3, and ciliary mutants rescued of their embryonic lethality by complementation with corresponding sense mRNAs (Grimes et al., 2016; Zhang et al., 2018) (Fig. 2A,B). MicroCT scans of these mutant fish revealed prominent abnormalities of their vertebral column (Fig. 2C–F), strongly similar to what has been described for Uts2r3 and cilia mutants (Grimes et al., 2016; Zhang et al., 2018). These findings establish that embryonic body curvature and scoliosis of the adult spine are linked events, and RF is not only required for proper development of the embryonic axis as reported previously (Cantaut-Belarif et al., 2018), but is also critically necessary for adult spine morphogenesis, implying its relevance to the etiology of IS.

### Expression of *urp* genes is strongly affected in *sspo* mutants

**Fig. 3.**
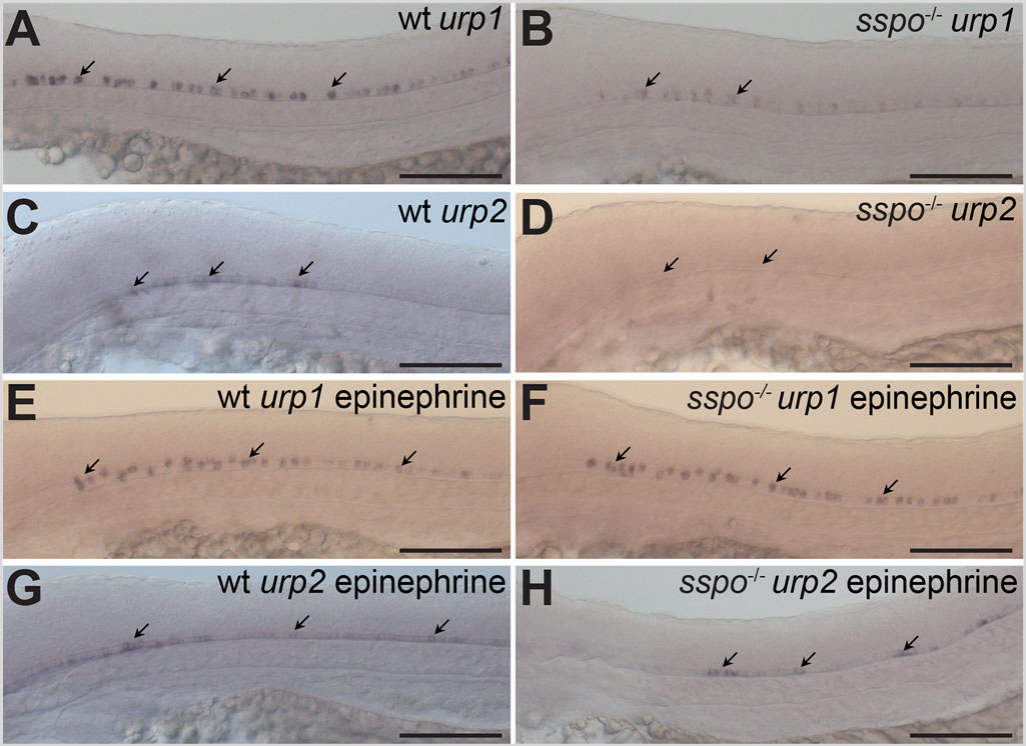
***sspo* mutants show loss of *urp* gene expression from CSF-cNs.** (A) A wild-type embryo, showing *urp1* expression in CSF-cNs (arrows). (B) An *sspo*-mutant embryo, showing reduction in levels of *urp1* expression from CSF-cNs (arrows). (C) A wild-type embryo, showing *urp2* expression in CSF-cNs (arrows). (D) An *sspo*-mutant embryo, showing almost complete lack of *urp2* expression from CSF-cNs (arrows). Embryos shown in A–D are representative of a minimum of 25 embryos analysed for each genotype for each *urp* gene. (E) A wild-type embryo, showing *urp1* expression in CSF-cNs after exposure to epinephrine (arrows). (F) An *sspo*-mutant embryo, showing *urp1* expression in CSF-cNs after exposure to epinephrine (arrows). Note that expression level is indistinguishable from wild type. Embryos from mating of *sspo*-heterozygous fish were exposed to epinephrine in two independent experiments (25 embryos each). Since all embryos showed similar levels of *urp1* expression, 13 embryos were randomly genotyped after *in situ* hybridisation from the second experiment, of which three were mutants. (G) A wild-type embryo, showing *urp2* expression in CSF-cNs after exposure to epinephrine (arrows). (H) An *sspo*-mutant embryo, showing restoration of *urp2* expression in CSF-cNs after exposure to epinephrine (arrows). Embryos from mating of *sspo*-heterozygous fish were exposed to epinephrine in two independent experiments (25 embryos each). 16 embryos from the second experiment, with *urp2* expression in the spinal cord, were genotyped after *in situ* hybridisation, of which five were mutants. All embryos depicted in A–H were at 26 hpf. Scale bars: 100 μm.

**Fig. 4.**
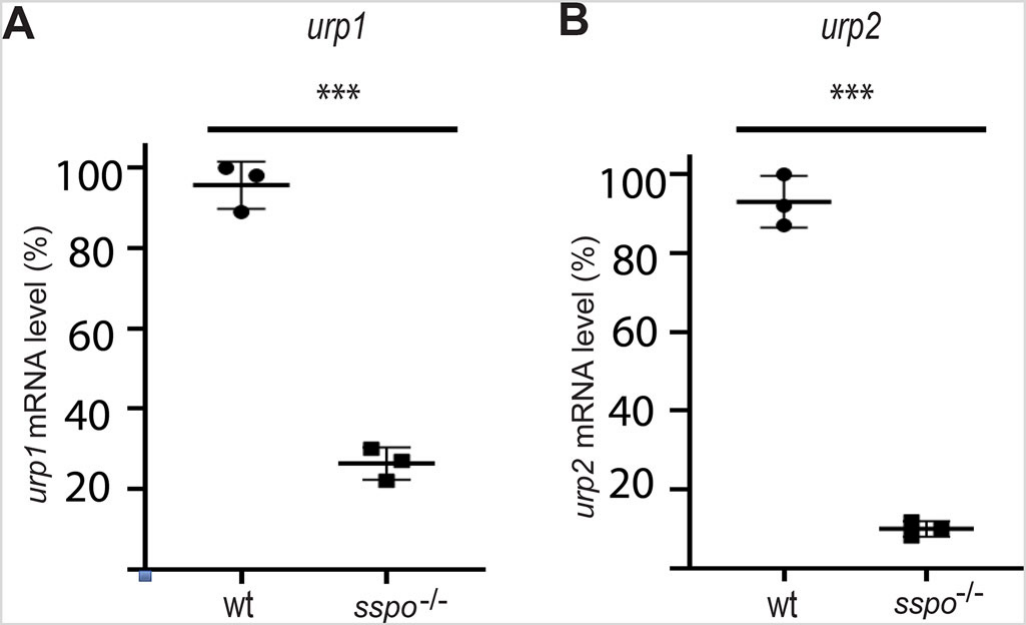
**RT-qPCR analysis of *urp1* and *urp2* expression in wild-type and *sspo* mutants.** (A) Expression level of *urp1* mRNA is reduced in *sspo* mutants relative to wild type. ***P=0.0004 [three independent experiments; for each experiment, mRNA from wild-type and *sspo* mutants was extracted from a pool of eight embryos (at 26 hpf) per genotype]. (B) Expression level of *urp2* is drastically reduced in *sspo* mutants relative to wild type. ***P=0.0001 (three independent experiments, for each experiment mRNA from wild-type and *sspo* mutants was extracted from a pool of eight embryos per genotype). Expression levels are plotted along the *y*-axis. The results were analysed using unpaired Student's *t*-test. Error bars represent standard deviation. Wt signifies mixture of wild-type and heterozygous siblings.

**Fig. 5.**
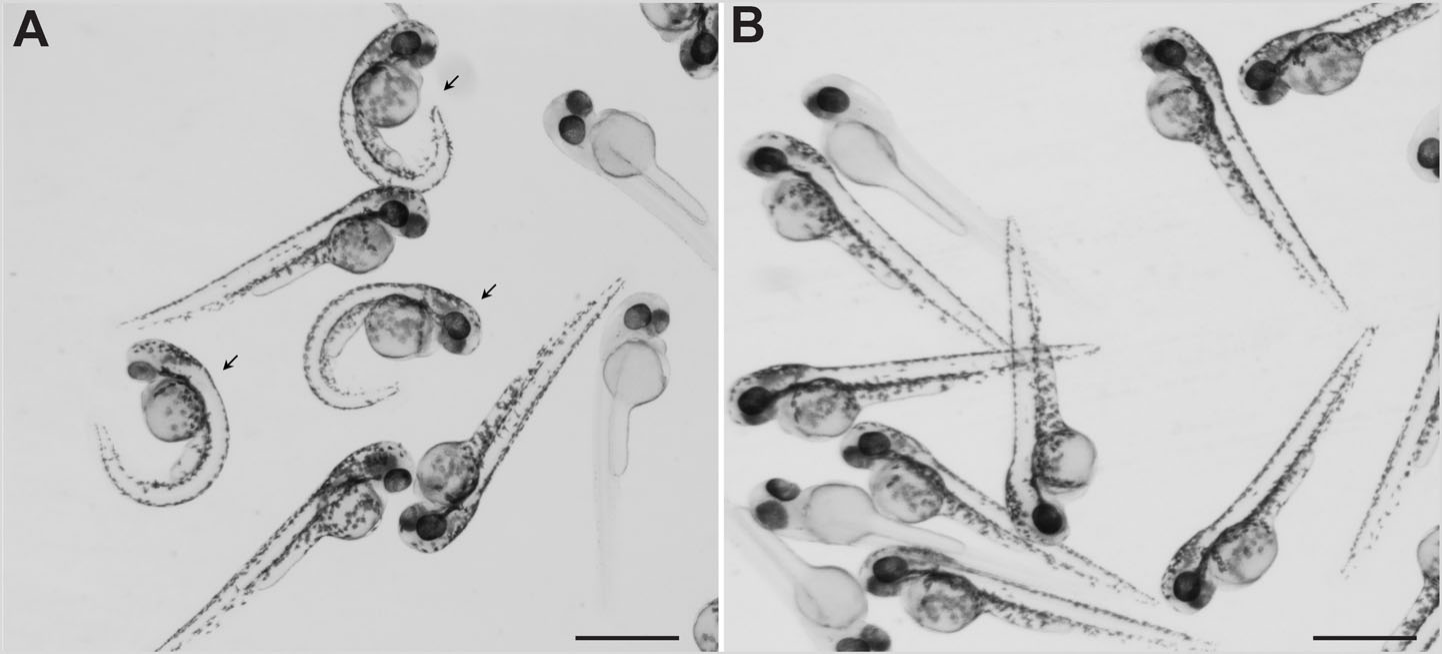
**Exposure to epinephrine rescued axial curvature of *sspo* mutants.** (A) *sspo* mutants (arrows) and siblings at 48 hpf. (B) *sspo* mutants and siblings at 48 hpf after epinephrine treatment. Note absence of embryos with curved bodies. Embryos shown in A and B are representative of a minimum of 100 embryos analysed in two independent experiments. Note, some embryos are devoid of body pigmentation as the *sspo* allele is maintained in the *nacre* mutant background that prevents body pigmentation in homozygotes. Scale bars: 1 mm.

RF is known to bind and transport catecholamines present in CSF (Caprile et al., 2003), but the significance of these properties has not been established. On the other hand, catecholamines, like epinephrine, seem to be the key factors in CSF that trigger expression of *urp* genes in CSF-cNs to bring about proper axial development (Zhang et al., 2018). Since ciliary motility is necessary for CSF flow as well as RF assembly (Cantaut-Belarif et al., 2018; Grimes et al., 2016; Zhang et al., 2018), development of axial defects in *sspo* mutants could be explained by their inability to bind epinephrine and present to CSF-cNs for activation of *urp* gene expression. We first examined the expression of *urp1* and *urp2*, two paralogous *urp* genes that encode cyclic peptides with supposedly similar activity, and which we have previously established to be expressed in CSF-cNs in response to epinephrine in the CSF (Zhang et al., 2018). Like in cilia motility mutants, we found that expression of *urp1* is reduced and *urp2* is almost completely absent in *sspo* mutants (Figs 3A–D and 4). Moreover, incubation of *sspo* mutants with epinephrine in the embryo culture medium from 16 hpf not only restored *urp* gene expression, but also rescued ventral curvature of their body axes (Figs 3E–H and 5).

### Restoration of Urp2 expression specifically in CSF-cNs of *sspo* mutants is sufficient to rescue embryonic and larval axial curvature and adult scoliosis

**Fig. 6.**
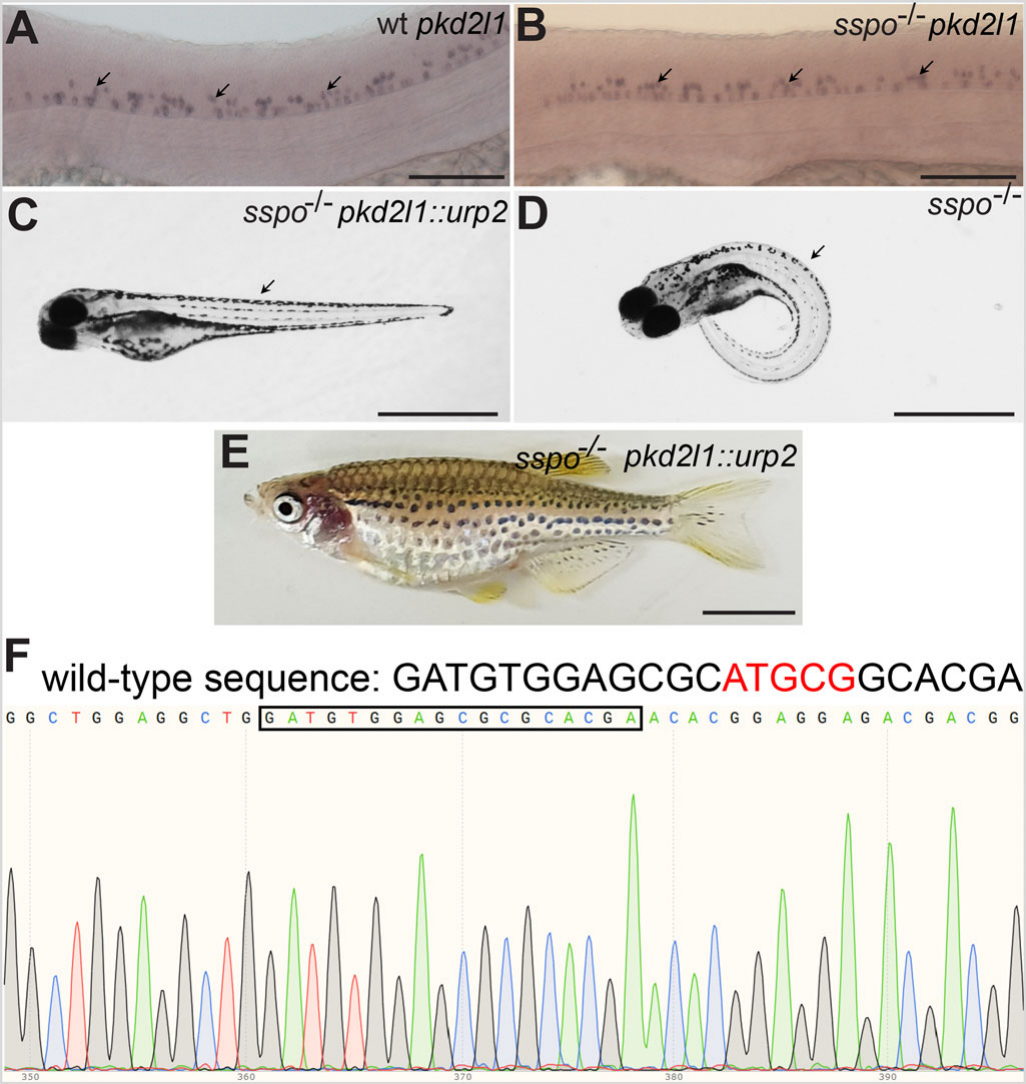
**Axial defects of *sspo* mutants can be rescued by restoring *urp2* expression specifically in CSF-cNs using the *pkd2l1* promoter.** (A) A wild-type embryo, showing *pkd2l1* expression in CSF-cNs (arrows). (B) An *sspo*-mutant embryo showing normal levels and pattern of *pkd2l1* expression in CSF-cNs (arrows). Embryos depicted in A and B were at 26 hpf and are representative of a minimum of 25 embryos analysed for each genotype. Scale bars: 100 μm. (C) An *sspo*-mutant larva rescued of axis curvature (arrow) by restoration of *urp2* expression using the *pkd2l1::urp2* transgene. A total of 150 embryos with straight axes from *sspo*/+ in-cross injected with *pkd2l1::urp2* transgene from two independent experiments were genotyped, and 20 were found to be *sspo*-homozygous mutants. (D) An *sspo*-mutant larva. Note the strong ventrally curved axis (arrow). The larvae depicted were at 5 dpf. Scale bars: 1 mm. (E) An *sspo*-mutant adult rescued of its scoliotic spine by restoration of *urp2* expression using the *pkd2l1::urp2* transgene. Of 92 adults with straight axes, 31 were wild type, 60 were heterozygous and one was a homozygous mutant. Scale bar: 0.5 cm. (F) Electropherogram showing homozygous mutant genotype of the rescued *sspo*-mutant adult depicted in E. The wild-type sequence is indicated on top, with the five bp that are deleted in the mutant in red. The mutant sequence (with five bp deletion) is highlighted with the black box.

To garner further evidence that it is indeed the loss of Urp signalling that is causative of the axial deformities in *sspo* mutants, we decided to restore Urp expression specifically in the CFS-cNs using transient transgenesis, and then assess for effects on body curvature. For this, we used the promoter of the *polycystic kidney disease 2-like 1* (*pkd2l1*) gene, which encodes a transient receptor potential channel expressed in the CSF-cNs (Böhm et al., 2016; Djenoune et al., 2014), to ensure as close to physiological levels of Urp2 expression as possible (see also Materials and Methods). We found that unlike the *urp* genes, *pkd2l1* expression is not discernibly affected in *sspo* mutants (Fig. 6A,B). Strikingly, *sspo* mutants expressing Urp2 under the control of the *pkd2l1* promoter showed significant rescue of embryonic body curvature, and many mutants developed into swimming larvae with straight axes, indistinguishable from their wild-type siblings (Fig. 6C), in contrast to the fully penetrant ventrally curved bodies of non-transgenic *sspo* mutants (Fig. 6D). We also screened a population of 3-month-old adult fish with straight body axes (these fish were derived from eggs obtained from *sspo*/+ heterozygous crosses injected with the *pkd2l1::urp2* transgene as described above for larval rescue), and found one homozygous mutant (Fig. 6E,F; cf. Fig. 2A,B). Thus, restoration of appropriate levels of Urp expression in CSF-cNs is sufficient for the rescue of axial deformities in *sspo* mutants (also see below and Discussion). Furthermore, these results provide strong evidence that it is indeed a deficiency in Urp signalling that is the unifying theme underlying the axial deformities of zebrafish deficient in cilia motility as well as RF.

### Chronic over-expression of Urp2 in somitic muscles induces upward curving of the body axis

**Fig. 7.**
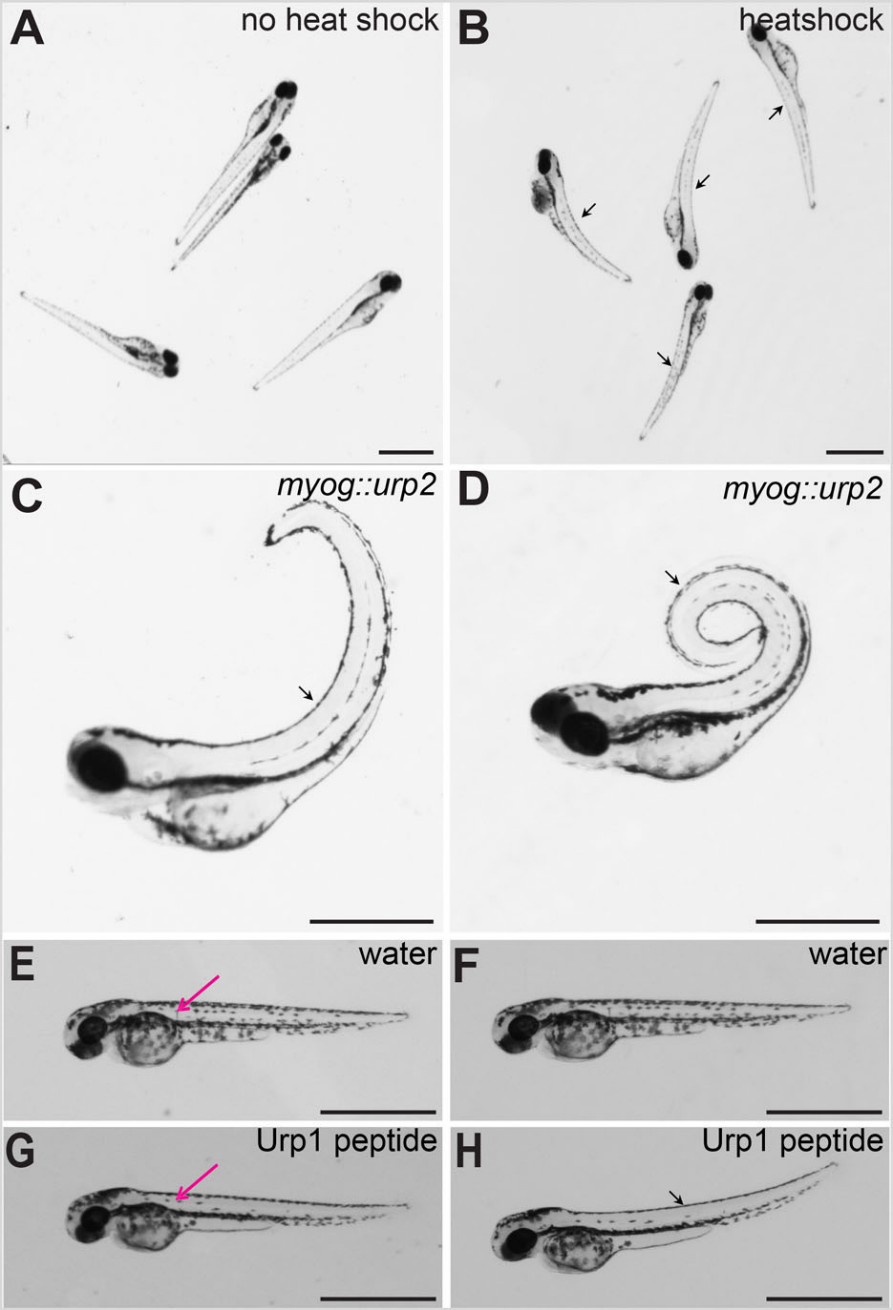
**Urp over-expression causes dorsal curvature of the body axis in zebrafish embryos.** (A) Wild-type embryos injected with *hs::urp2* transgene, without heat induction. Note no effect on the body axis. (B) Same batch of embryos imaged after 30 min post-heat shock. Note the initiation of dorsal curvature of the body axis in the heat-shocked embryos (arrows). (C) A wild-type embryo at 72 hpf injected with *myog::urp2* transgene, showing dorsal curvature of the trunk and tail (arrow). (D) A wild-type embryo at 5 dpf injected with *myog::urp2* transgene showing spiral coiling of the body axis (arrow). Embryos shown in A–D are representative of a minimum of 100 embryos analysed for each condition of Urp2 over-expression, in two independent experiments. (E) A wild-type embryo at 48 hpf, injected with water in the dorsal somite, imaged immediately after injection. (F) The same wild-type embryo depicted in E, imaged after 1 h. Note no effect on body axis position. (G) A wild-type embryo at 48 hpf, injected with synthetic Urp1 in the dorsal somite, imaged immediately after injection. (H) The same wild-type embryo depicted in G, imaged after 5 min. Note the dorsal curvature of the body axis (arrow). In E and G, the injection sites are indicated (magenta arrows). Four embryos were injected for each condition. Scale bars: 1 mm.

Since somitic muscle cells have been postulated to be the target of Urp action (Zhang et al., 2018), we next modulated Urp signalling globally using the heat-inducible promoter (Halloran et al., 2000), or locally in the muscle cells themselves using the skeletal muscle-specific *myogenin* (*myog*) promoter (Srinivas et al., 2007). Wild-type embryos injected with the heat-shock inducible *urp2* transgene showed no abnormalities in their body axis in the absence of heat induction (Fig. 7A). Remarkably, a 15 min, 37°C heat-shock-induced expression of Urp2 led to a rapid response apparent in the upward (dorsal) curvature of the body axis (Fig. 7B). This effect was a temporary deformation of the trunk and tail, as maintenance of the heat-shocked embryos at normal growth temperature (28.5°C) following the heat-shock treatment allowed them to recover and regain the straight body axis characteristic of control embryos. Wild-type embryos not injected with the heat-shock *urp2* transgene, but subjected to the same heat-shock regime, showed no discernible effect on body axis positioning (data not shown).

In contrast to heat-shock promoter mediated Urp2 over-expression, constitutive expression of Urp2 in the somitic muscle cells using the *myog* promoter induced a permanent upward curvature of the body axis that gradually intensified with time (Fig. 7C). The entire axis not only curved dramatically upwards, but in many of the larvae it was also thrown into a spiral coil as development progressed (Fig. 7D). Similar effects of *myog* promoter-driven expression of Urp2 were also observed in *sspo* mutants (data not shown). We have previously reported that injection of synthetic Urp1 into the brain ventricles of zebrafish embryos was sufficient to rescue the body curvature of cilia mutants and to induce upward body curvature in wild-type siblings (Zhang et al., 2018). To provide additional evidence that muscle cells are indeed the target of Urp activity, we directly injected Urp1 into the trunk musculature of 48 hpf embryos. As with the heat-shock induced pulse of Urp2 expression, these embryos responded instantaneously with upward curvature of their body axis (Fig. 7E–H). Again, similar to heat-shock driven Urp2 expression, the effect was transitory and the injected embryos recovered their straight axes over time.

### Slow-twitch muscle fibre deficient embryos are refractory to Urp over-expression

**Fig. 8.**
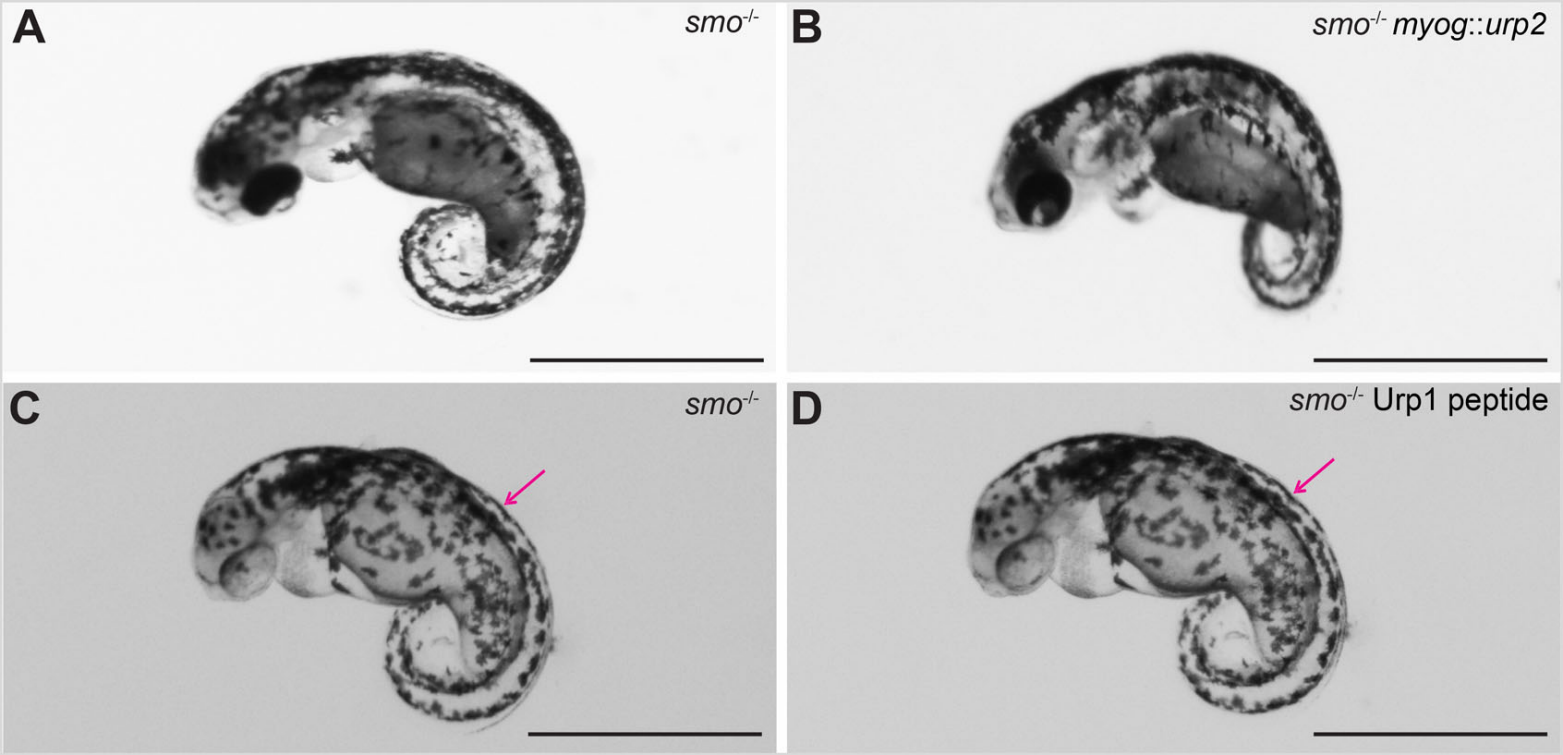
***smo*-mutant embryos, lacking slow-twitch fibres, are unresponsive to Urp over expression.** (A) An *smo*-mutant embryo at 48 hpf. (B) An *smo*-mutant embryo injected with *myog::urp2* transgene. Note that there was no effect on the ventrally curved axis. This experiment was performed in two independent biological replicates. In the first batch, five wild-type siblings showed no dorsal curvature and 57 showed dorsal curvature. All 12 *smo* mutants showed no response. In the second batch, of 105 wild-type siblings all showed dorsal curvature. All 24 *smo* mutants showed no response. (C) An *smo*-mutant embryo at 48 hpf, injected with water in the dorsal somite, imaged after 1 h. (D) An *smo*-mutant embryo, injected with Urp1 in the dorsal somite, imaged after 1 h. Note that there was no effect on the ventrally curved axis. The injection sites are indicated (magenta arrows). Four embryos were injected for each condition. Scale bars: 1 mm.

Zebrafish trunk musculature broadly comprises two kinds of fibre types, slow twitch and fast twitch (Jackson and Ingham, 2013). In our earlier study, we demonstrated that Uts2r3, the relevant receptor for Urps in axial morphogenesis, is expressed in slow-twitch fibres of the dorsal somite, implicating the slow-twitch muscles as effectors of Urp signalling (Zhang et al., 2018). To provide additional evidence that it is the slow-twitch muscles that respond to Urp signalling and regulate axis development, we used the *myog* promoter, which is active in both the slow and fast-twitch lineages, to express Urp2 in embryos mutant for the *smoothened* (*smo*) gene. Like cilia and *sspo* mutants, *smo*-mutant embryos exhibit profoundly curved body axes (Barresi et al., 2000; Chen et al., 2001), which arises from loss of motile cilia (and likely RF) from the brain and spinal cord, as well as the CSF-cNs themselves, since Smo participates in Hedgehog (Hh) signalling to specify and pattern the ventral brain and spinal cord, from which ciliated cells and CSF-cNs are derived (Zhang et al., 2018). In addition, *smo* mutants lack the slow-twitch muscle cells from their somites, as Hh activity is also required to specify this muscle cell-type (Barresi et al., 2000). If slow-twitch muscles are indeed necessary for responsiveness to Urp activity, then *smo* mutants should be refractory to the over-expression of Urp. Consistent with this view, *myog*-promoter-driven over-expression of Urp2 failed to elicit any changes in their strong, ventrally curled body axis, unlike the dorsal curvature that could be induced in wild-type embryos and *sspo* mutants (Fig. 8A,B). Finally, we also found that direct injection of Urp1 into the trunk musculature failed to induce any alteration in the body curvature of *smo* mutants (Fig. 8C,D), further confirming that it is the slow-twitch fibres that mediate the effects of Urp signalling on musculoskeletal coordination for proper morphogenesis of the body axis.

## DISCUSSION

**Fig. 9.**
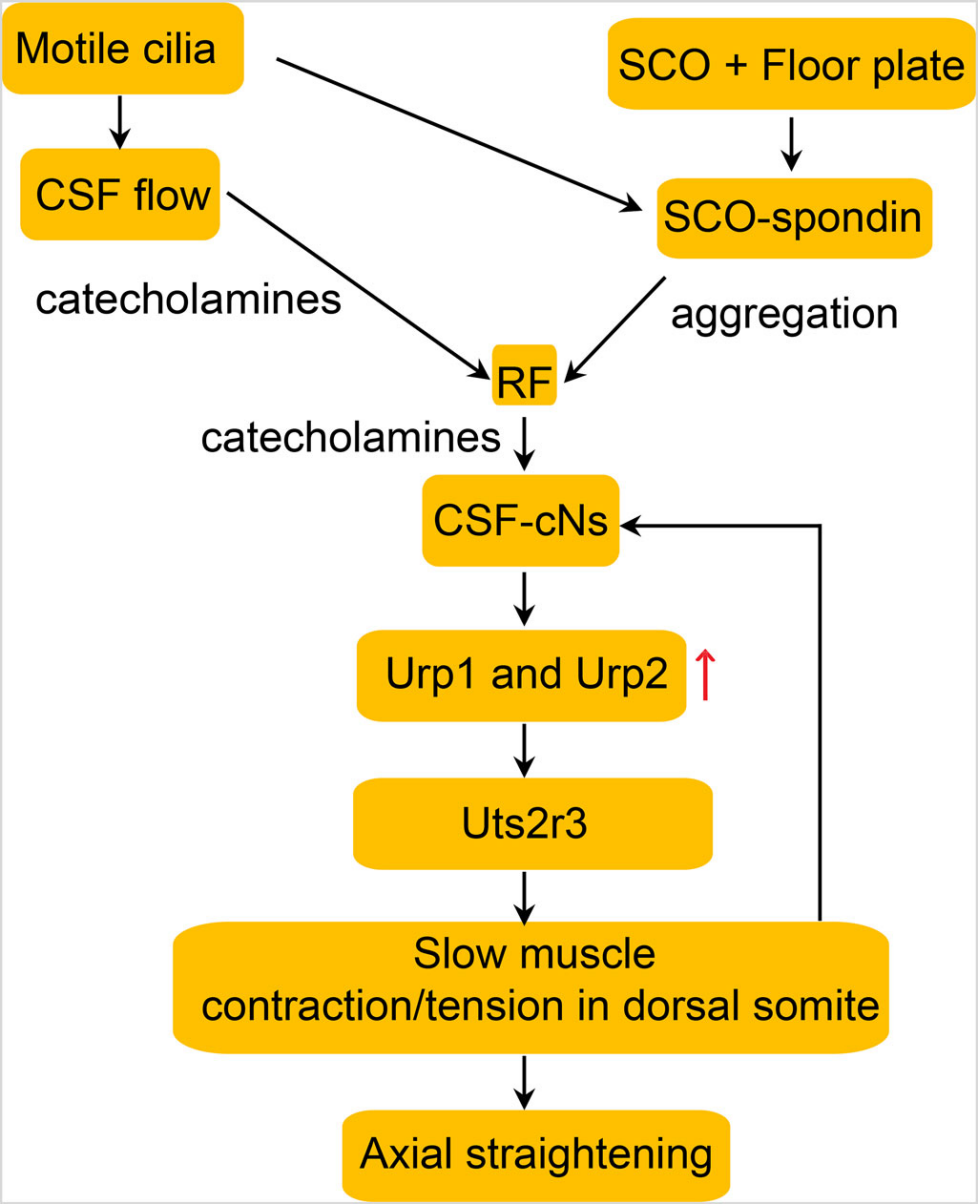
**Model of how cilia, CSF flow, RF and CSF-cNS regulate body-axis straightening in the zebrafish via Urp signalling and slow-twitch muscle activity.**

For more than a century, the biological functions of RF have been a topic of considerable intrigue and speculation. Although recently RF has been shown to be important for proper development of the body axis in the zebrafish embryo, the molecular mechanism involved was completely unexplored (Cantaut-Belarif et al., 2018). We have now shown that loss of RF not only affects the embryonic axis, but also causes scoliosis of the spine in adult zebrafish, and reconciled that impairment in cilia-driven CSF flow, as well as the loss of RF, impact axial development through a common mechanism of derailing the Urp-signalling pathway (Fig. 9). Scoliosis has been reported to be more prevalent among patients with cilia motility defects as well as individuals with Parkinson's disease, which affects the catecholamine synthesising neurons of the brain, (Baik et al., 2009; Engesaeth et al., 1993) and it is also a co-morbid clinical feature among people affected with muscular dystrophies and myopathies (Claeys, 2020), underscoring the relevance of skeletal muscle dysfunction in spine malformations. Furthermore, given the very large size of the gene, which makes it susceptible for accumulation of deleterious variants, it is likely that mutations in *SSPO* could underlie the development of IS in some of the familial and sporadic cases of the disease. This prediction is in line with the reported observation that RF is present in human embryos and in teenagers, which coincides with the onset of IS symptoms during early childhood and adolescence (Cheng et al., 2015; Olry and Haines, 2003).

Urp receptors are G-protein coupled receptors, and there is abundant experimental evidence that they signal by mobilising intracellular Ca^2+^ and can induce spasmogenic effects on many tissues and cell-types (Vaudry et al., 2015). These findings are consistent with the model that Urp-mediated contraction or tension in the slow-twitch fibres of the dorsal somites provides the critical biomechanical cue for proper alignment of the embryonic axis and the adult spine during development. Besides secreting Urp peptides, CSF-cNs also project to spinal circuits involved in locomotion and posture in the zebrafish, and can sense mechanical bending and longitudinal contractions of the trunk and tail via the Pkd2l1 channel (Knafo and Wyart, 2018; Orts-Del'Immagine and Wyart, 2017; Sternberg et al., 2018). This implies a feedback loop between the CSF-cNs and axial muscles that is likely to continually modulate muscle activity through Urp signalling to direct proper axial development (Fig. 9). In line with this view, the central finding that has emerged from our earlier (Zhang et al., 2018) and present analysis is that a specific threshold of Urp signalling is the critical determining factor for proper directionality of axial morphogenesis. In fact, it was only with a low dose of transient expression of Urp2 under the control of the *pkd2l1* promoter, specifically in the CSF-cNs, that we succeeded in obtaining effective rescue of the body curvature of the *sspo* mutants, although the low frequency of rescue all the way to adulthood that we observed represents an obvious limitation of this strategy. In transient transgenesis, since the transgene is not stably integrated in the genome, there is a considerable degree of variability in the duration and levels of expression, as well as the ability to target the cell-type in question in sufficient numbers, likely causing a significant number of *sspo* homozygotes, which were rescued through embryonic development to subsequently perish during larval and juvenile stages. Higher doses of injection of the *pkd2l1::urp2* transgene led to increasing numbers of embryos with dorsal curvature of the axis, as we observed with the heat-shock and *myog* promoter transgenes (data not shown), implying that even if stable transgenesis with *pkd2l1::upr2* were to be attempted to attain a higher degree of rescue of *sspo* mutants to the adult stage, the levels of transgene-driven Urp expression will have to be quite precisely controlled to achieve rescue and prevent the over-expression phenotype of dorsal curvature. In sum, all of these findings provide several lines of evidence that there is indeed a definite measure of Urp-signalling level that is critical for proper axial morphogenesis: optimal level ensures a straight axis, loss of signalling produces a ventrally curved axis, while exaggerated signalling can bring about the converse effect of profound dorsal curvature.

Although IS is a relatively common disorder, treatment options for this condition are rather limited and often require major surgical alterations of the vertebral column. Since our data show that direct manipulation of Urp signalling in the trunk musculature can elicit immediate changes in axial positioning in the zebrafish, therapeutic interventions for managing and rectifying spinal deformities in IS could potentially be derived from appropriate pharmacological exploitation of this pathway.

## MATERIALS AND METHODS

### Zebrafish strains

All zebrafish strains were maintained according to standard procedures for fish husbandry. The following wild-type and mutant strains were used in this study: AB, inbred wild-type control; *sspo^icm13^*, a null allele of *sspo* with five base pair (bp) deletion in the second coding exon (Cantaut-Belarif et al., 2018) and *smo^hi1640^*, a retroviral insertional mutation in *smo* gene (Chen et al., 2001). All experiments with zebrafish were approved by the Singapore National Advisory Committee on Laboratory Animal Research.

### Raising *sspo* mutants to adulthood

Embryos derived from crosses of *sspo^icm13^* heterozygous parents were manually dechorionated at 24 hpf. The homozygous mutants could be identified by their characteristic curled-down body axis. Mutants and siblings were then cultured separately using standard procedure for raising fry to adulthood.

### Wholemount *in situ* hybridisation and qPCR analysis

Wholemount *in situ* hybridisation with digoxigenin labelled *urp* and *pkd2l1* gene riboprobes, described earlier (Zhang et al., 2018), was done following routine protocol. Using the EXPRESS SYBR GreenER Super Mix kit (Invitrogen, A10315), qPCRs were performed on an Applied BioSystems 7900HT Fast Real-Time PCR System with the SDS2.4 software. For each genotype, technical triplicate reactions were performed. mRNA-expression-level differences between any two samples were calculated from the Ct values after normalising against mRNA for the internal control, *gapdh*.

### MicroCT imaging

Adult *sspo* mutants and wild-type siblings were euthanised using an overdose of Tricane and eviscerated. The carcasses were fixed in 4% paraformaldehyde over night at 4°C, dehydrated through grades of ethanol (4 h for each grade) and then into 100% ethanol with overnight incubation. MicroCT images were acquired using an Inveon CT (Siemens AG, Berlin, Germany) at 55 kVp/110 mA. The exposure time per projection was 2500 ms and a binning factor of 2 was used, resulting in a reconstructed pixel size of 35 µm. Planar images were acquired from 181 projections over 360° of rotation. The images were reconstructed using a Feldkamp cone-beam algorithm. Three-dimensional renders of the skeleton were made with AMIRA software (FEI, France) with constant window settings. Raw data were viewed with AMIDE 1.0.4 software (Sourceforge), and quantified using Fiji software (ImageJ).

### Generation of *pkd2l1::urp2*, *hs::urp2* and *myogenin::urp2* transgenes

The *pkd2l1::urp2* construct was generated by cloning *pkd1l1* promoter (3 kb region before the *pkd2l1* start codon) and *urp2* coding sequence into pEGFP1 vector with *Hind*III and *Not*I restriction enzyme sites. The *hs::urp2* construct was generated by cloning zebrafish *urp2* coding sequence into HspIG vector with *BamH*I and *Not*I restriction enzyme sites. The *myog::urp2* construct was generated by cloning *myog* promoter region (800 bp region before the *myog* start codon) and *urp2* coding sequence into pEGFP1 vector with *Xho*I and *BamH*I restriction enzymes.

### Microinjection of *urp2* transgenes into eggs for transient transgenesis

Plasmids with *urp2* transgenes under the control of different promoters were linearised, and the DNA was injected into fertilised eggs obtained from wild-type or *sspo* heterozygote fish in-crosses at a concentration of 30–50 ng/µl, 0.5 nl per egg. The injected eggs were then cultured to the desired developmental stages. For the *pkd2l1::urp2* transgene, a higher dose of injection led to upward curvature of the body axis like the heat-shock and *myog* promoter constructs, which is why we used the 30–50 ng/µl, 0.5 nl per egg dose for all rescue experiments.

### Heat-shock mediated induction of Urp2

*hs::urp2*-transgene-injected and uninjected control embryos were heat shocked for 15 min by immersion of the culture flask in a 37°C water bath. After heat shock, the embryos were cultured at the normal growth temperature of 28.5°C and imaged at several time points to record alterations in their body axes.

### Intramuscular injection of Urp1

Intramuscular injection of synthetic Urp1 (800 µg/ml in water and 0.5 nl per embryo) or water (0.5 nl per embryo) was performed unilaterally in the dorsal somite (typically somite number 5–6) of 48 hpf wild-type and *smo*-mutant embryos anesthetised with Tricane. The embryos were imaged before and at several time points after injection.

### Epinephrine treatment

Embryos derived from crosses of *sspo* heterozygous parents were incubated with 10 mg/ml epinephrine (Sigma-Aldrich, E4642) in embryo medium from 16 hpf until 26 or 48 hpf. Following treatment, the embryos were dechorionated and imaged or fixed and processed for *in situ* hybridisation.

### Genotyping

PCR and Sanger sequencing-based genotyping was used to unambiguously identify homozygous *sspo* mutants, heterozygous and wild-type siblings in epinephrine treatment and Urp over-expression experiments. Primers for genotyping have been described before (Cantaut-Belarif et al., 2018).

### Microscopy

Embryos were imaged either using a Leica stereomicroscope (M 205 FA) fitted with a Leica camera (DFC 7000 GT) or a Zeiss compound microscope (Imager. Z1) fitted with a Zeiss camera (AxioCam HRc).

### Figure assembly

All figures were assembled using Adobe Illustrator CS4.
